# Novel folliculin gene mutations in Polish patients with Birt–Hogg–Dubé syndrome

**DOI:** 10.1186/s13023-021-01931-0

**Published:** 2021-07-06

**Authors:** Elżbieta Radzikowska, Urszula Lechowicz, Jolanta Winek, Lucyna Opoka

**Affiliations:** 1 3rd Department of Lung Diseases and Oncology, National Tuberculosis and Lung Diseases Research Institute, Warsaw, Poland; 2 Department of Genetics and Clinical Immunology, National Tuberculosis and Lung Diseases Research Institute, Warsaw, Poland; 3 Outpatient Department, National Tuberculosis and Lung Diseases Research Institute, Warsaw, Poland; 4 Department of Radiology, National Tuberculosis and Lung Diseases Research Institute, Warsaw, Poland

## Abstract

**Background:**

Birt–Hogg–Dubé syndrome (BHDS) is a rare, autosomal dominant, inherited disease caused by mutations in the folliculin gene *(FLCN)*. The disease is characterised by skin lesions (fibrofolliculomas, trichodiscomas, acrochordons), pulmonary cysts with pneumothoraces and renal tumours. We present the features of Polish patients with BHDS.

**Materials and methods:**

The first case of BHDS in Poland was diagnosed in 2016. Since then, 15 cases from 10 families have been identified. Thirteen patients were confirmed via direct *FLCN* sequencing, and two according to their characteristic clinical and radiological presentations.

**Results:**

BHDS was diagnosed in 15 cases (13 women and 2 men) from 10 families. The mean ages at the time of first pneumothorax and diagnosis were 38.4 ± 13.9 and 47.7 ± 13 years, respectively. Five patients (33%) were ex-smokers (2.1 ± 1.37 packyears), and 10 (67%) had never smoked cigarettes. Twelve patients (83%) had a history of recurrent symptomatic pneumothorax. Three patients had small, asymptomatic pneumothoraces, which were only detected upon computed tomography examination. All patients had multiple bilateral pulmonary cysts, distributed predominantly in the lower and middle, peripheral, and subpleural regions of the lungs. Generally, patients exhibited preserved lung function. Skin lesions were seen in four patients (27%), one patient had renal angiomyolipoma, and one had bilateral renal cancer. Different mutations of the *FLCN* gene were identified (mainly in exon 6), with two novel heterozygous variants: c.490delA p.(Arg164GlyTer13) and c.40delC p.(His14ThrsfTer41).

**Conclusions:**

All analysed patients with BHDS presented with lung lesions and with less frequent skin and renal lesions than previously reported in other populations. In addition, more frequent mutations located in exon 6 were detected, and two novel *FLCN* gene mutations were identified.

**Supplementary Information:**

The online version contains supplementary material available at 10.1186/s13023-021-01931-0.

## Introduction

Birt–Hogg–Dubé syndrome (BHDS) is a rare, autosomal dominant, inherited disease caused by mutations in the folliculin gene (*FLCN*) located on the short arm of chromosome 17p11.2. *FLCN* functions as a tumour suppressor gene. The protein product of this gene, folliculin, participates in the proper functioning of the cellular cytoskeleton and extracellular matrix as well as proliferation. Expression of *FLNC* is observed in the lung, skin, distal nephrons, brain, heart, placenta, and testis [1–3]. Therefore, affected patients present with lesions in these organs [1, 4–6].

The disease is characterised by skin lesions (fibrofolliculomas, trichodiscomas, acrochordons) that usually occur on the face, neck and upper torso, pulmonary cysts with pneumothoraces, and renal tumours. BHDS was first described more than 50 years ago, and since then, approximately 600 families with the syndrome have been identified worldwide [4, 5, 7–9].

A wide spectrum of BHDS symptoms has been found in different populations, and phenotypic variability is seen, even within the same family. Usually, the first manifestation of the syndrome is skin lesions, which develop at the age of 20–30 years in > 80–90% of patients [2, 9–12]. Subsequently, pneumothorax, as a typical feature of lung involvement, presents at the age of 30–40 years; 50–60% of patients experience pneumothorax, which is usually recurrent. Various sizes, shapes and numbers of thin-walled cysts, located in the middle and lower parts of the lungs and in the subpleural regions, are frequently observed in approximately 90% of BHDS patients [8, 9, 13–15]. Generally, lung function is preserved, and even in patients with multiple cysts, there is no respiratory insufficiency[14].

Renal tumours occur, usually in the fifth decade of life in < 30% of patients, and sometimes this is an isolated presentation of the disease [16–18]. Patients with BHDS develop various kinds of renal tumours, often differentiated within the same organ. The most frequently identified tumours are hybrid oncocytic tumours with features of both chromophobe renal cell carcinoma (RCC) and oncocytoma, chromophobe RCC, and clear cell RCC [6, 8–11].

To date, more than 200 mutations in the *FLCN* gene have been identified and documented in the FLCN Leiden Open Variation Database. The most frequently detected mutations are protein truncations, including frameshift (small deletions or insertions), nonsense or splice variants. The detection rate of *FLCN* mutations in patients with BHDS features is approximately 90% [7, 14, 19–28].

We present the clinical, radiological and genetic features of Polish patients with BHDS.

## Materials

From 2016 to 2020, 15 patients with BHDS have been diagnosed in our department. The diagnosis, based on criteria proposed by the European BHD Consortium, was established when patients fulfilled at least one major or two minor criteria. The major criteria were at least five fibrofolliculomas or trichodiscomas (at least one histologically confirmed, of adult onset) and a pathogenic *FLCN* germline mutation. The minor criteria were multiple lung cysts (bilateral basally located lung cysts with no other apparent cause, with or without a spontaneous primary pneumothorax), renal cancer (early onset before age 50 y, multifocal or bilateral, mixed chromophobe, and oncocytic histology) and a first-degree relative with BHDS [27].

Fibrofolliculomas and trichodiscomas were diagnosed by dermatological evaluation.

Chest computed tomography (CT) scans were evaluated in all patients for the presence of pneumothorax, cysts and cyst localisation (central part of both lungs), cyst size (maximum diameter: < 6 mm, > 15 mm), cyst shape (round, oval) and lung involvement (25%, 50% and 75% of lung fields).

Pulmonary function tests (PFTs) were performed according to the joint guidelines of the American Thoracic Society and European Respiratory Society, at 3–6 months after an episode of pneumothorax. Lung volumes were measured using body plethysmography (Jaeger MasterScreen software ver. 4.65; Würzburg, Germany), and the diffusion capacity of the lungs for carbon monoxide (T_L_,_CO_) was determined using the single-breath technique. The predicted values were analysed.

CT or magnetic resonance imaging of the abdomen was used for abdominal assessment.

*FLCN* gene detection was performed based on methods described in the literature. Genomic DNA was extracted from peripheral blood leukocytes and subjected to polymerase chain reaction and Sanger sequencing. The sequencing reactions were conducted on an ABI 3730 DNA Analyzer (Thermo Fisher Scientific, Waltham, MA, USA), and the resulting chromatograms were analysed using Variant Reporter 1.1 software (Applied Biosystems, Thermo Fisher Scientific) [11].

In addition, for two patients, next-generation sequencing reactions were performed on a HiSeq 4000 Sequencer (Illumina Inc., San Diego, CA, USA) with a reading length of 2 × 151 nucleotides and an average coverage of 230 × with a quality threshold of 100%. Libraries were prepared using the enrichment method. Genetic variants were identified using Burrows-Wheeler Aligner. The method allows detection of 100% of substitutions and 95% of small deletions and insertions. The coding sequence of the *FLCN* gene was analysed along with 10–20-nucleotide intron flanks.

The pathogenic variant nomenclature presented follows the Human Genome Variation Society recommendations (https://varnomen.hgvs.org/; version 20.05). DNA mutation numbering is based on the *FLCN* cDNA sequence (GenBank accession numbers NM_144997.7) with the A of the ATG translation‐initiation codon numbered as + 1. Similarly, amino-acid numbering starts with the translation initiator methionine as + 1 (NP_659434.2).

The study was approved by the Ethics Committee of the National Tuberculosis and Lung Diseases Research Institute (Number 36/2020) and performed according to the tenets of the Declaration of Helsinki.

## Results

BHDS was diagnosed in 15 patients from 10 families. Thirteen patients were confirmed by direct sequencing of the *FLCN* gene and two by clinical diagnosis. Three patients have been reported previously [29, 30].

The mean ages at the time of first pneumothorax and diagnosis were 38.4 ± 13.9 and 47.7 ± 13 years, respectively. Five patients (33%) were ex-smokers (2.1 ± 1.37 packyears), and 10 (67%) had never smoked cigarettes.

Skin lesions were seen in four (27%) patients, but none had undergone histological verification. The mean age at first lesion detection was 28.7 ± 1.2 years.

One patient (7%) was diagnosed with bilateral renal cancer at the age of 47 years, one had renal angiomyolipoma (AML), and renal cysts were detected in three (20%) patients (Table 1).

**Table 1:** Patients characteristics

Family	Patient	Sex	Age at diagnosis ( years)	Age at first pneumothorax (years)	Lung	Skin	Kidney	Smoking (pack/ years)	Pneumothora× symptomatic	Asthma	Others	Other cancers	Benign tumours
1	1.1	M	45	30	Yes	Yes	cyst	0	3	No	Hypothyroidism psoriasis	No	No
2	2.1	W	26	26	Yes	No	No	0	0	Yes	No	No	No
	2.2	W	60	48	Yes	No	No	0	3	Yes	Arterial hypertension	No	No
	2.3	M	36	31	Yes	No	No	5	4	Yes	No	No	No
3	3.1	W	68	66	Yes	No	AML, cyst	4	0	Yes	Arterial hypertension	No	Myoma uteri, endometriosis
4	4.1	W	54	34	Yes	Yes	No	1,5	4	No	No	No	Breast fibroadenoma, myoma uteri
5	5.1	W	50	30	Yes	Yes	No	0	0	No	No	No	Ovarian cyst
	5.2	W	44	34	Yes	No	No	0	3	Yes	Arterial hypertension	No	No
6	6.1	W	62	47	Yes	No	Bilateral kidney cancer	5	5	No	No	No	Myoma uteri
7	7.1	W	35	20	Yes	Yes	No	0	5	No	No	No	No
8	8.1	W	54	43	Yes	No	No	0	2	No	Yes	Ovarian cancer	No
	8.2	W	27	21	Yes	No	No	0	3	No	No	No	No
9	9.1	W	52	50	Yes	No	cyst	2	0	No	Hypothyroidism	No	
10	10.1	W	40	34	Yes	No	cyst	0	3	No	No	No	Endometriosis
	10.2	W	62	62	Yes	No	No	0	2	No	No	No	
Mean (SD)			**47.7 ± 12.96**	**38.4 ± 13.9**				**2,1 ± 1,37**	**2.46 ± 1.76**				
All					**15 (100%)**	**4 (27%)**	**2 (12%)**		**11 (70%)**	**5 (33%)**		**1 (7%)**	

AML—renal angiomyolipoma, No—no lesion was detected, Yes—presence of lesions

### Pneumothoraxes

Thirty-seven symptomatic episodes of pneumothorax were observed, which usually treated with pleural derange. The mean number of pneumothoraxes was 2.46 ± 1.76. Video-assisted thoracoscopy was performed in 6(40%) patients bilaterally and in 6(40%) cases unilaterally. Persistent air leakage resulted in subsequent surgical procedure in 3(20%) patients. In addition, mechanical pleurodesis, chemical pleurodesis and pleurectomy were done in 17, 1, and 6 patients respectively. In two patients, after pleurodesis and pleurectomy 3 episodes of recurrent pneumothorax were noticed, all of them underwent conservative treatment.

### Concomitant diseases

Three (20%) patients had a history of uterine leiomyoma, two (13%) were diagnosed with endometriosis, one had a breast fibroadenoma, one had an ovarian cyst, and in one-woman, ovarian cancer was diagnosed at the age of 29 years. Asthma was diagnosed in five (33%) patients from three families, hypothyroidism in two (13%) patients, and arterial hypertension in three (20%) patients (Table 1).

### Family history

In six families, 12 additional possible cases with features of BHDS were reported (Table 2), mainly skin lesions and/or pneumothoraxes. In addition, two fathers of patients had lung cancer, another one had an oral cavity cancer, and one was diagnosed with lymphoma.

**Table 2:** Number of affected family members and cancer cases in the first-degree relatives

Family	Patient	Number of other affected family members	Other cancers in family members
1	1.1	1 (Father)	Lung cancer—father
2	2.1		
	2.2		
	2.3		
3	3.1	2 (Sister and her son)	
4	4.1	2 (Grandmother, sister of mother)	
5	5.1		Lymphoma—father
	5.2		
6	6.1	1(Father renal cancer)	
7	7.1	4 (Father, fathers brother and his son, sister of father)	
8	8.1		Lung cancer -father
	8.2		
9	9.1	2 (Mother and sister pneumothoraxes)	
10	10.1		
	10.2		Oral cavity cancer
All		12	4

### Genetic analyses

Different mutations of the *FLCN* gene were identified. Mutations in exon 6 were found in four families, and in exons 4, 9, 11 and 13 in one family each. Single nucleotide deletions and three-nucleotide deletions were detected in four and two families, respectively. Substitution of cytosine to thymine at position 499 of the coding sequence of the *FLCN* gene was found in one family.

Novel heterozygous sequence variants of c.490delA p.(Arg164GlyTer13) were detected in two unrelated families in exon 6. This deletion cause frameshift and premature STOP codon. In addition, deletion c.40delC p.(His14ThrsfTer41) was identified in exon 4. These mutations have not been reported previously in any of the HGMD (Human Gene Mutations Database), Ensembl and ClinVar databases (Table 3).

**Table 3:** Mutations characteristics

Family	Patient	Mutation	Exon	Genotype	Genotype compatible with HGVS v.2 (on the level of cDNA)	Genotype compatible with HGVS v.2 (on the level of protein)
1	1.1	c40delC	4	His14fsTrfsTer41/−	NM_144997:c40delC	NP_659434.2: p[His14fsTrfsTer41]
2	2.1	c.490delA	6	Arg164GlyTer13/−	NM_144997.5: c[490delA]	NP_659434.2: p[Arg164GlyTer13]
	2.2					
	2.3					
3	3.1	c.499C > T	6	p.Glyn167Ter/−	NM_144997.5: c.[499C > T]	NP_659434.2: p[Gln167Ter]
4	4.1	c.469_471delTTC	6	c.469_471delTTC/−	NM_144997.5: c.[469_471delTTC]	NP_659434.2: p.[Phe157del]
5	5.1	c.1285delC	11	p.His429TherTer39	NM_144997.5:c.1285delC	NP_659434.2: p.[His429TherTer39]
	5.2					
6	6.1					
7	7.1					
8	8.1	c.1522_1524 delAAG	13	p.Lys508del/−	NM_144997.5: c.[1522_1524delAAG]	NP_659434.2: p.[.Lys508del]
	8.2					
9	9.1	c490delA	6	Arg164GlyTer13/−	NM_144997.5: c[490delA]	NP_659434.2: p[Arg164GlyTer13]
10	10.1	c987delC	9	Ser330fs	NM_144997: c[987delC]	NP_659434.2: p[Ser330fs]
	10.2					

### Radiological examinations

Twelve patients (83%) had a history of recurrent symptomatic pneumothorax. Three patients had small, asymptomatic pneumothoraxes, which were detected only in CT scans. All patients had multiple bilateral pulmonary cysts. Lesions involving < 25% of lung fields were observed in 26% of the patients, but in 60% of the BHDS patients, cysts were seen in 75% of the lung fields. Both small and large cysts were visualised, but in two (13%) patients, only very small lesions were detected. Distribution throughout both lungs was seen in 67% of the patients, but in the central regions only in 50% of the patients (Table 4). (Additional file 1: Fig. S1–Additional file 6: Fig. S6.)

**Table 4:** Characteristics of pulmonary lesions revealed by chest computed tomography (CT)

Family	Patient	Lesions in central part of lungs	Lesions in all lungs	Small cysts 6 mm	Cyst > 15 mm	Small and large cysts	Lesions in 1/4 of lungs	Lesions in 1/2 of lungs	Lesions in > 3/4 of lungs	Pneumothorax on CT examination
1	1.1		1			1			1	Yes
2	2.1	1		1			1			Yes
	2.2	1		1				1		Yes
	2.3	1			1		1			Yes
3	3.1		1			1			1	Yes
4	4.1		1			1			1	Yes
5	5.1		1			1			1	Yes
	5.2		1			1			1	Yes
6	6.1	1			1			1		Yes
7	7.1	1		1			1			Yes
8	8.1	1				1			1	Yes
	8.2		1			1	1			Yes
9	9.1		1			1			1	Yes
10	10.1		1			1			1	Yes
	10.2		1			1			1	Yes
All	12	6	9	3	2	9	4	2	9	15

### Pulmonary biopsy

Pulmonary biopsy specimens were available for nine patients who underwent video-assisted thoracoscopy with pleurodesis. Histological assessment showed thin-walled cysts, which were lined by cuboidal epithelium without signs of fibrosis or smooth muscle cells in the wall (Additional file 7: Fig. S7).

### Pulmonary function tests

In one older woman (68 y), pulmonary function parameters were slightly decreased (forced expiratory volume in 1 s [FEV1]: 68% predicted and T_L,CO_: 60% predicted); in all other patients the forced vital capacity and FEV1 were within normal limits. Two (14%) patients had > 120% the predicted value of total lung capacity (TLC), and eight (53%) patients had a residual volume > 120% (Table 5).

**Table 5:** Pulmonary function parameters in patients with BHDS ( VC, vital capacity; FEV1,-force

Family	Patient	VC% pred	VC (L)	FEV1 (%pred)	FEV1 (L)	TLC (%pred)	TLC (L)	RV (%pred))	RV(L)	Tl,_co_	Tl,_co_ (%pred)	TVPG (mmHg)
1	1.1	85	3.83	87	3.1	86	5.62	86	1.79	7.77	81	21
2	2.1	120	3.5	110	2.5	No	No	No	No	No	No	No
	2.2	121	3.6	103	2.5	No	No	No	No	No	No	No
	2.3	110	4.1	100	3.6	No	No	No	No	No	No	No
3	3.1	80	1.94	68	1.3	96	4.06	133	2.43	3.75	60	28
4	4.1	90	3.0	91	2.4	104	5.22	121	2.22	5.65	81	21
5	5.1	91	3.0	86	2.4	103	5.57	129	2.42	8.08	93	No
	5.2	86	3.15	102	2.58	102	5.55	86	1.53	9.25	102	No
6	6.1	102	3.65	95	2.35	116	5.65	132	2.55	5.97	80	26
7	7.1									No	No	No
8	8.1	116	3.37	116	2.7	123	6.2	127	2.3	5.88	73	21
	8.2	120	4.99	120	4.19	129	6.88	130	1.89	6.99	72	No
9	9.1	102	3.63	99	2.83	112	5.84	120	2.21	6.78	81	22
10	10.1	96	3.65	86	2.69	113	5.79	133	2.3	7.49	85	21
	10.2											
Mean (SD)		102.0 ± 15.1	3.45 ± 0.69	96.9 ± 14.6	2.68 ± 0.66	108.4 ± 12.8	5.64 ± 0.7	119.7 ± 18.33	2.16 ± 0.32	6.76 ± 1.53	80.8 ± 11.48	22.86 ± 2.9

In family 2 and in one patient from family 10, PFTs were not performed, because at the time of the appointments they had experienced recent pneumothoraxes.

### Echocardiography

No patients (of 7 examinations performed) had any echocardiographic evidence of pulmonary hypertension, and the pulmonary arterial pressures were within normal limits.

## Discussion

This study presents the first population of Polish patients with BHDS. All cases had cystic lung lesions with pneumothoraxes, which were recurrent in all patients with symptomatic pneumothorax. Skin lesions were identified in 27%, and renal changes in 17% of the patients. The lung was the only organ involved in 50% of the patients. The most frequent mutations were in exon 6. In two families, a novel heterozygous sequence variant of c.490delA p.(Arg164GlyTer13) in exon 6, and in one family, a novel c.40delC p.(His14ThrsfTer41) mutation in exon 4 were identified.

The prevalence of specific organ lesions in BHDS differs in relation to the cohorts previously reported by a dermatologist, nephrologist and pulmonologist [1, 7, 16, 18, 30, 31].

Pneumothorax was reported in 42–88% of pulmonary cohorts and in 23–38% of renal/dermatological cohorts. Recurrent pneumothorax was reported in 75–80% of BHDS patients [8, 12], with an average of > 3 episodes [8]. Houweling et al. reported on a group of 115 Dutch patients from 35 families and found a history of pneumothorax in 28% of patients, which was recurrent in 80%, with a mean age of first pneumothorax of 36 years, similar to our group [32]. We want to emphasise the value of chest CT examination in the detection of pulmonary lesions, even in patients without symptoms. This allowed us to diagnose small asymptomatic pneumothoraces in 20% of the cases.

Similar to our group, Liu et al. observed pulmonary cystic lesions in all Chinese patients enrolled, with less frequent skin fibrofolliculomas (10%) and renal tumours (22%) [33]. Of 12 Korean patients, 100%, 18% and 9% presented with pulmonary, skin and renal manifestations of the disease, respectively [34].

In our group, renal cysts were diagnosed in three (20%) patients, and it has been suggested that this may be the only manifestation of the disease [7, 9, 17]. Generally, renal tumours are detected in > 30% of patients by the mean age of 50 years [6, 17]. Of note, our patient developed bilateral clear cell renal cancer, at an earlier age, before the age of 50 years. Zabar et al. evaluated the risk of developing renal cancer in patients with BHDS in comparison with their unaffected family members and disclosed a seven-fold greater risk of kidney tumour, and 50-fold higher risk of pneumothorax [6]. In addition, Nahorski et al. found a higher risk of colon neoplasms in BHDS patients but van de Beek et al. analysed 399 Dutch patients with BHDS, and an increased risk of colon carcinoma was not reported [35, 36]. However, in this study, patients with BHDS underwent removal of colon polyps more frequently, but the number of patients with polyps was similar to that of unaffected family members. Similar observations were presented by Zabar et al., who also did not find a predisposition to colon polyps in BHDS patients [6]. Thyroid nodules, lipomas, liver cysts and parotid oncocytomas have been reported in BHDS patients [6, 35, 36]. However, no previous data presented such a high prevalence of gynaecological tumours (ovarian cancer, leiomyoma uteri, endometriosis, ovarian cyst) as we found in our group of patients. This observation warrants future studies in larger patient populations.

There is a wide spectrum of *FLCN* gene mutations. The gene is located on chromosome 17p11.2 and consists of 14 exons, of which 11 are coding exons [9]. In our study, substitution and deletion mutations were identified, resulting in nonsense and frameshift protein changes, leading to loss of folliculin function. Structurally destabilised, truncated variants of the FLCN protein are degraded by a proteasome [37–39]. The *FLCN* gene has been implicated in the pathogenesis of BHDS and its role as a tumour suppressor gene is well established. Toro et al. revealed *FLCN* mutations in 81% (154/190) of patients and 85% (68/80) of BHDS families [12]. However, the lack of a detected mutation in the *FLCN* gene does not exclude the disease.

In another study, Toro et al. reported that in 17 (48%) patients and 47% of families, mutations were identified in exon 11, which suggested this region as a mutation ‘hot spot’ for BHDS [11]. Similar observations were reported by Nickerson et al., Nahorski et al., Furuyama et al., and Liu et al. [1, 33, 35, 40]. Of note, in our group of BHDS patients, a mutation in exon 11 was found in only one family and detected mutations were most frequently located in exon 6. This may suggest the specific regional appearance of the mutational profile, but further study of a larger group is required. In addition, two novel mutations were found in three families. Furuya et al. investigated the mutation spectrum and clinicopathologic findings of 312 patients from 120 different families (119 Japanese and 1 Taiwanese) [19]. They identified 31 different *FLCN* sequence variants; almost all patients presented with lung lesions, 13% with renal lesions, and 49% with skin lesions.

Recently, a high number of new mutations was identified by Liu et al. in a Chinese population; of 20 mutations, 14 were novel [33].

A clear association between the disease phenotype and the type of mutation has not been established. Toro et al. noticed that mutations in exon 9 were associated with an increased number of cysts, and mutations in exon 9 and 12 with a higher number of pneumothoraces, but in our group, we did not observe such a correlation [12].

Knowledge about this rare disease and awareness of BHDS by pulmonary physicians are increasing. In Poland, the first case of BHDS was diagnosed in 2016; subsequently, 14 other cases have been identified [29, 30]. We noticed a rather long mean diagnosis delay of approximately 20 years between first symptom appearance and the diagnosis of BHDS. None of our patients were referred due to suspicion of the disease. Four of these patients had been observed for many years at our Institute, with a diagnosis of lymphangioleiomyomatosis, pulmonary Langerhans cell histiocytosis or emphysema. Most lung lesions had been diagnosed as emphysema, supported not only by an incorrect radiological assessment but also by histological assessment of the lung samples.

The size of the group is the most important limitation of this study. BHDS is a very rare disease, and the symptoms are often wrongly associated with other diseases. However, even in such a small group, two novel *FLCN* gene mutations have been discovered.

Future studies are needed to assess the prevalence of the disease in the Polish population and to clarify possible linkages to gynaecological tumours.

## Supplementary Information

**Additional file 1**:** Figure S1**. Chest CT, lung window, coronal plane. Patient 2.3. There is a left sided pneumothorax and cysts in left lung.

**Additional file 2**:** Figure S2**. Patient 2.3. Reconstruction in 3D Pulmo software depicts areas of less than -950HU in colour red.

**Additional file 3**:** Figure S3**. Chest CT, lung window, coronal plane. Patient 6.1. Computed tomography scan shows characteristic distribution of lung cysts predominantly in lower lung zones.

**Additional file 4**:** Figure S4**. Patient 6.1. Reconstruction in 3D Pulmo software depicts areas of less than -950HU in colour red.

**Additional file 5**:** Figure S5**. Chest CT ,lung window, axial plane. Patient 3.1. Computed tomography scan shows characteristic distribution of lung cysts predominantly in lower lung zones, on the left side pleural thickening are present.

**Additional file 6**:** Figure S6**. Chest CT ,lung window, axial plane. Patient 2.2. Computed tomography scan shows small lung cysts in lower lung zones.

**Additional file 7**:** Figure S7**. Patient 2.3. Histological assessment showed thin-walled cysts, which were lined by cuboidal epithelium ( H&E staining, low magnification). Curtesy of the Professor R. Langfort.

## Data Availability

All source data are presented in the article.
