# Supplementary figures and images for: Novel folliculin gene mutations in Polish patients with Birt–Hogg–Dubé syndrome
Source: Orphanet J Rare Dis. 2021 Jul 6;16:302. doi: 10.1186/s13023-021-01931-0 (PMC8258955; doi:10.1186/s13023-021-01931-0)

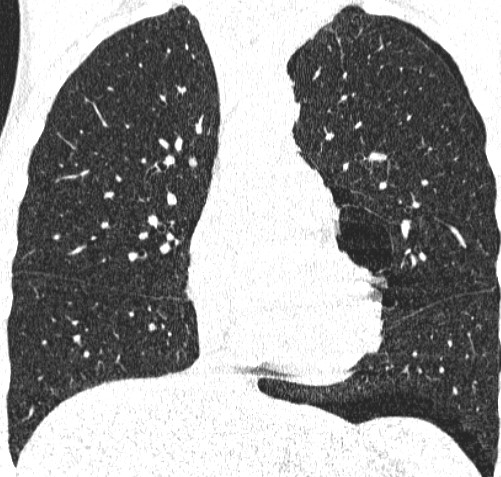

Supplement: Supplementary file 1 — Additional file 1: Figure S1. Chest CT, lung window, coronal plane. Patient 2.3. There is a left sided pneumothorax and cysts in left lung. [file 13023_2021_1931_MOESM1_ESM.jpg]

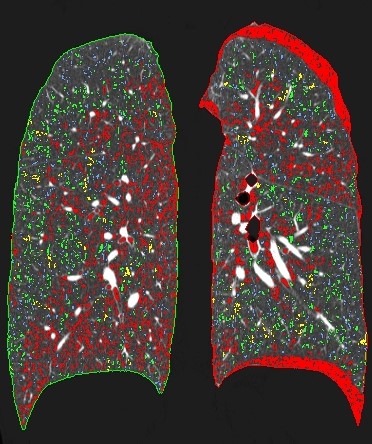

Supplement: Supplementary file 2 — Additional file 2: Figure S2. Patient 2.3. Reconstruction in 3D Pulmo software depicts areas of less than -950HU in colour red. [file 13023_2021_1931_MOESM2_ESM.jpg]

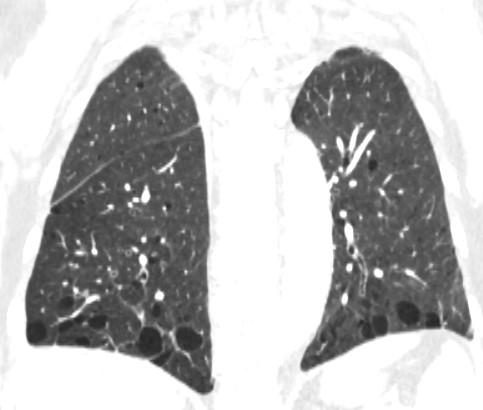

Supplement: Supplementary file 3 — Additional file 3: Figure S3. Chest CT, lung window, coronal plane. Patient 6.1. Computed tomography scan shows characteristic distribution of lung cysts predominantly in lower lung zones. [file 13023_2021_1931_MOESM3_ESM.jpg]

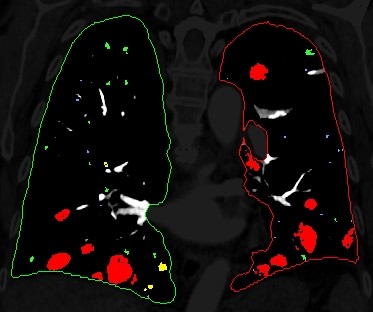

Supplement: Supplementary file 4 — Additional file 4: Figure S4. Patient 6.1. Reconstruction in 3D Pulmo software depicts areas of less than -950HU in colour red. [file 13023_2021_1931_MOESM4_ESM.jpg]

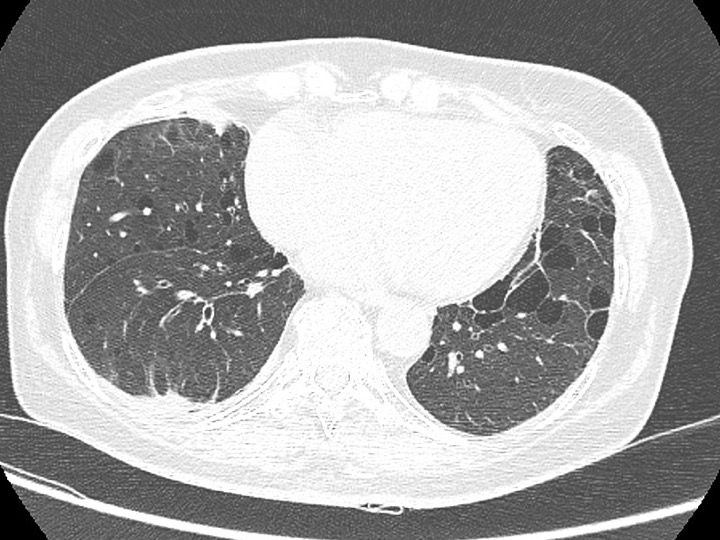

Supplement: Supplementary file 5 — Additional file 5: Figure S5. Chest CT ,lung window, axial plane. Patient 3.1. Computed tomography scan shows characteristic distribution of lung cysts predominantly in lower lung zones, on the left side pleural thickening are present. [file 13023_2021_1931_MOESM5_ESM.jpg]

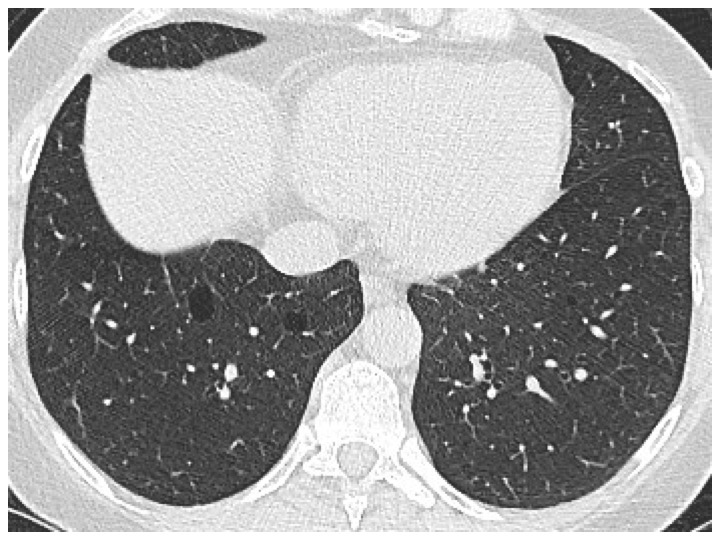

Supplement: Supplementary file 6 — Additional file 6: Figure S6. Chest CT ,lung window, axial plane. Patient 2.2. Computed tomography scan shows small lung cysts in lower lung zones. [file 13023_2021_1931_MOESM6_ESM.jpg]

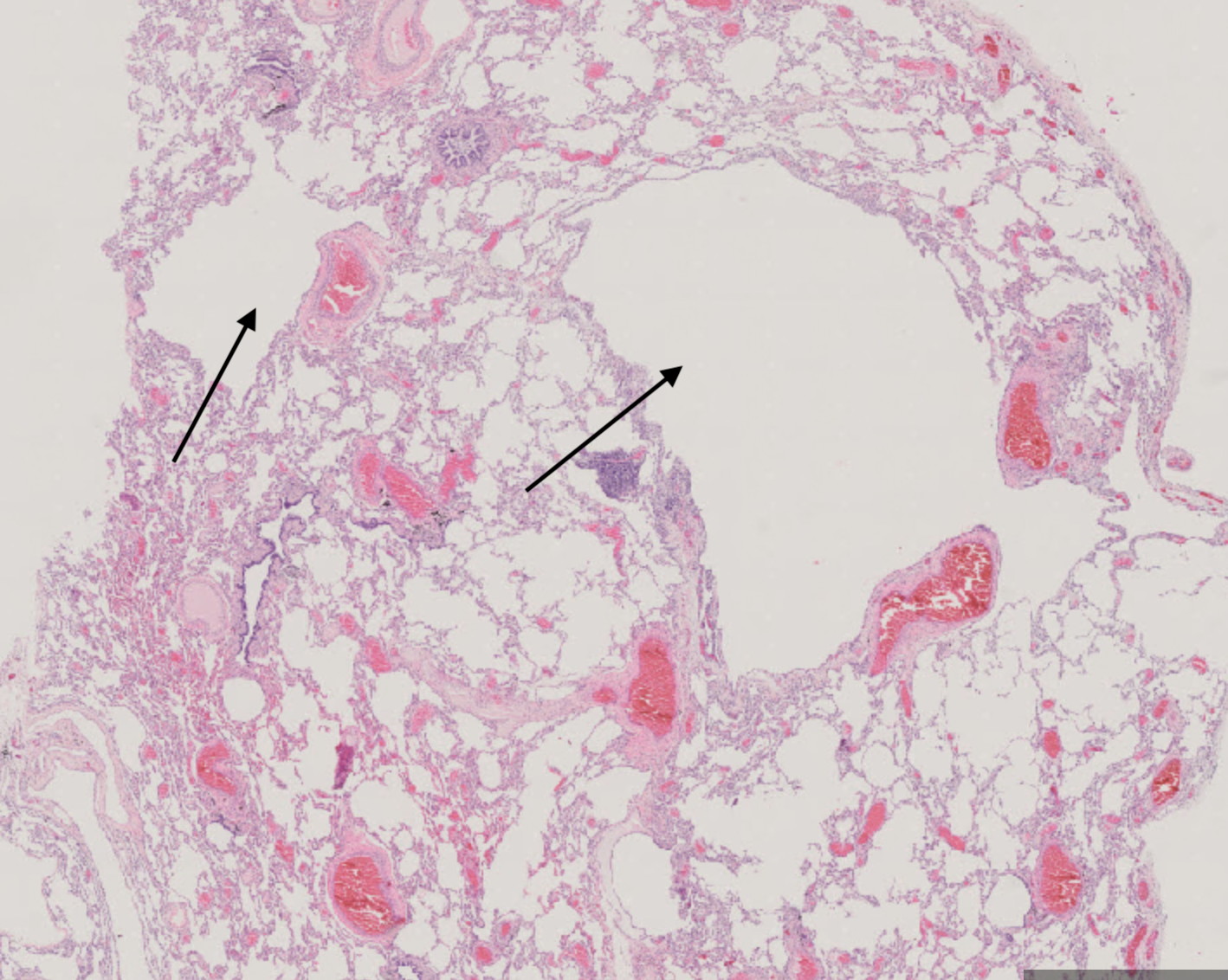

Supplement: Supplementary file 7 — Additional file 7: Figure S7. Patient 2.3. Histological assessment showed thin-walled cysts, which were lined by cuboidal epithelium ( H&E staining, low magnification). Curtesy of the Professor R. Langfort. [file 13023_2021_1931_MOESM7_ESM.tif]
